# Zenker’s diverticulum in an 85-year-old Ugandan man

**DOI:** 10.1186/s12876-021-01914-2

**Published:** 2021-08-30

**Authors:** Senai Goitom Sereke, Felix Bongomin, Zeridah Muyinda

**Affiliations:** 1 Department of Radiology and Radiotherapy, School of Medicine, Makerere University College of Health Sciences, Kampala, Uganda; 2 Department of Medicine, School of Medicine, Makerere University College of Health Sciences, Kampala, Uganda; 3 Department of Medical Microbiology and Immunology, Faculty of Medicine, Gulu University, Gulu, Uganda; 4 Department of Radiology, Mulago National Referral Hospital, Kampala, Uganda

**Keywords:** Zenker’s, Diverticulum, Barium swallow, Videofluoroscopy, Case report

## Abstract

**Background:**

Zenker’s diverticulum (ZD) is an uncommon disorder due to an outpouching of tissue through the Killian triangle that is thought to be caused by dysfunction of the cricopharyngeal muscle.

**Case presentation:**

An 85-year-old male patient presented with odynophagia and dysphagia of initially solid food followed by fluids that was associated with a significant weight loss over a one-year period. Barium swallow videofluoroscopy demonstrated a posterior outpouching of proximal esophagus that was 2 cm from the epiglottis. With the diagnosis of medium sized ZD, the patient underwent endoscopy guided diverticulotomy. Six months after the procedure, he was asymptomatic and had gained weight.

**Conclusions:**

Dysphagia and weight loss raises a clinical suspicion for a malignancy. Barium swallow examination is an inexpensive method for the diagnosis of ZD.

## Background

Zenker’s diverticulum (ZD), is a rare disorder with an estimated prevalence ranging between 0.01 and 0.11% in the general population [1]. It is an acquired outpouching of the mucosal and submucosal layers originating in a region of relative weakness known as Killian’s triangle or Killian’s dehiscence [2]. The most common pathogenesis relates to increased intraluminal pressure leading to an outpouching in an area of relative wall weakness when compared with surrounding tissues [3]. This area of weakness is located in the hypopharynx between two strong pharyngoesophageal muscles, the cricopharyngeus muscle and inferior pharyngeal constrictor [4]. It occurs between the seventh and eighth decade of life and rarely before the age of 40 [5]. It occurs with aging specifically due to changes in fibrosis and muscle necrosis of the upper esophageal sphincter [6]. Symptoms may present for weeks to years before presentation and diagnosis. Most patients present with a complaint of dysphagia [7]. Two mechanisms have been proposed by which the diverticulum can cause dysphagia: incomplete opening of the upper esophageal sphincter and extrinsic compression of the cervical esophagus by the diverticulum [8]. As the diverticulum enlarges, the symptoms become more severe with resultant weight loss. A sudden increase in severity of hematemesis may signal the development of ulcer [9]. Esophagography is necessary to confirm the diagnosis of ZD [1]. Several surgical options are available for the management of ZD [10]. Herein, a typical case of ZD diagnosed through barium swallow examination is reported in an elderly man.

## Case presentation

An 85-year-old male was referred to our center with a history of pain in swallowing and later regurgitation of undigested contents to his mouth 3-4 h after meals. The odynophagia and dysphagia was progressive in the last one-year before his presentation. Initially his dysphagia was to solid food alone and later to both solid foods and fluids. His appetite was affected by his odynophagia and he lost weight in 6-month period, in which he or his family members failed to quantify. There was no history of halitosis. One month prior to his presentation, the patient developed intermittent cough during meals. He was relatively healthy prior to onset of these symptoms. There was no history of hypertension or diabetes mellitus.

On physical examination, he was visibly wasted however his vitals were normal and there were no neck swelling or enlarged neck lymph nodes. His abdomen was soft and non-tender and no organomegaly was detected. His complete blood count showed hemoglobin concentration of 10.9 gm/dl, total white blood cell count of 7000/ml and platelets of 250,000/µl.

**Fig. 1:**
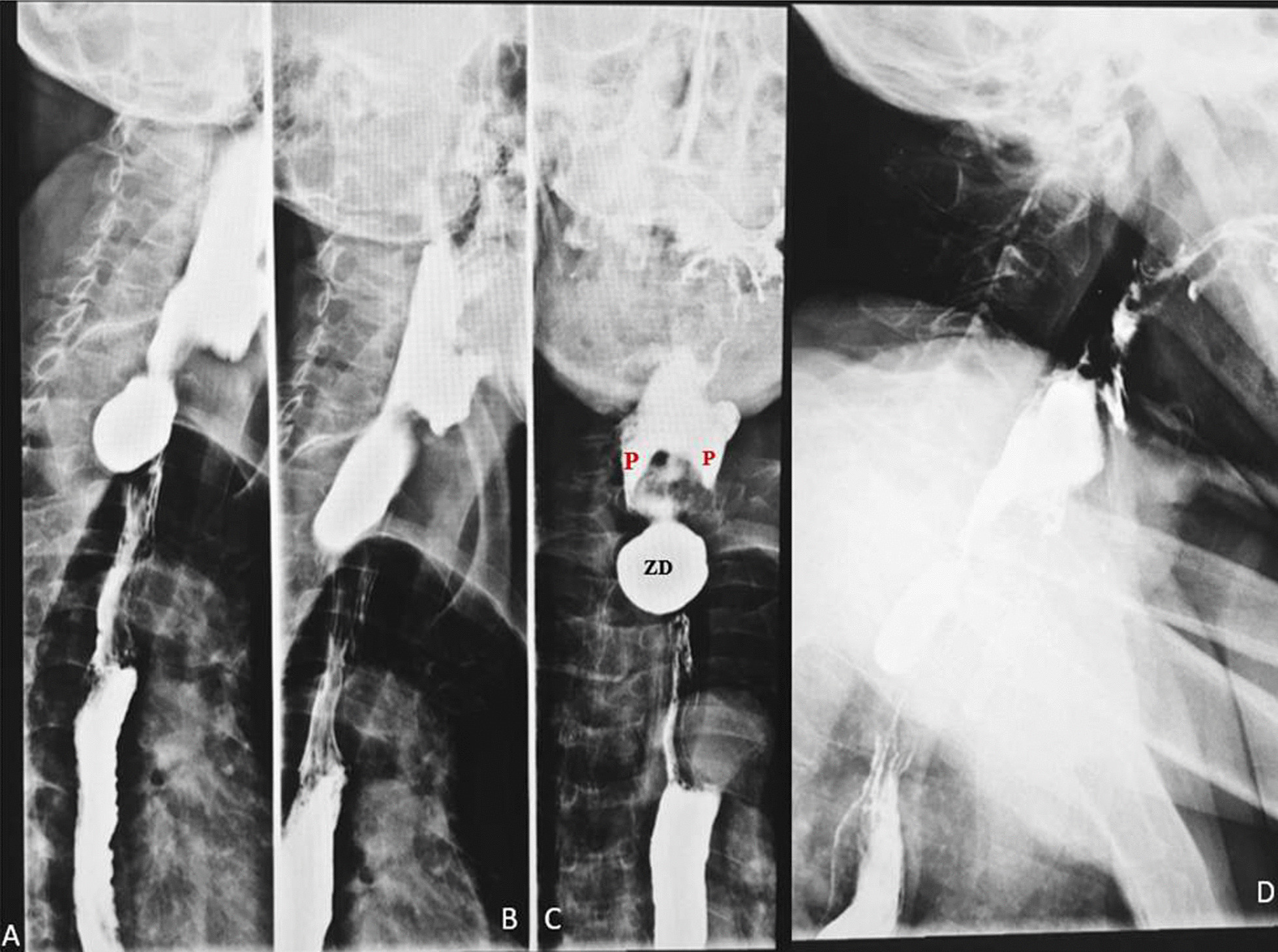
Static radiographic images taken from Videofluoroscopic esophagram **A** RAO view demonstrates posterior oval outpouching of the proximal esophagus with normal contrast flow in to the distal esophagus. **B** Between RAO and right lateral view demonstrated an elongation of the outpouching with additional contrast. **C** AP view demonstrated the dilated pyriform recess (P) and the diverticulum (ZD) **D** Lateral view demonstrates contrast coated pharynx and hypopharynx, pyriform and vallecullae

Esophagogram was done with oral suspension of barium sulphate demonstrated a smooth posterior outpouching of the proximal esophagus which was 2 cm from the epiglottis. The outpouching demonstrated an air-contrast level and measured 4.2 × 3.4 × 3 cm. However, normal propulsion of contrast was demonstrated below the outpouching. There was dilatation of the vallecullae and piriform recesses with temporary upholding of barium sulphate suspension (Fig. 1). The mid and distal esophagus was normal in caliber and mucosal lining. The tertiary waves were normal on dynamic examination but not demonstrated in the post processed images. So on the basis of the location of lesion and pouch like appearance; it was diagnosed as ZD.

The patient was assessed by a surgeon and gastroenterologist and flexible endoscopy guided diverticulotomy was performed. There were no immediate or late post-operative complications. During a 6 months follow up, his symptoms had completely resolved and he added some weight. The patient said that he was satisfied by the treatment he received.

## Discussion and conclusion

Esophageal diverticula are rare, with a radiologic and endoscopic prevalence of 0.06–4%, in which Zenker’s is the most common [11]. It is usually diagnosed between the seventh and eighth decades of life [5]. In this case, the patient presented in his mid-eighties.

The commonest symptoms of ZD at presentation are dysphagia, regurgitation of undigested food, choking, borborygmi in the cervical region, chronic cough, halitosis, weight loss, and less commonly hoarseness [12]. The most consistent sign, however, is dysphagia. Physical examination finding that is rarely seen is a swelling in the neck that my gurgle on palpation (Boyce’s sign). In the reported cases, there is variable time of presentation, from onset to diagnosis, ranging from weeks to years [6, 12]. The patient presented with odynophagia and dysphagia initially to solid and later to fluids. Then he developed regurgitation of undigested food. There were complaints of weight loss but no signs of malnutrition. There was no palpable neck swelling on examination and the duration of symptoms at presentation was only one year.

Barium swallow is the most important diagnostic tool for dysphagia. Contrast video fluoroscopy allows constant monitoring of the swallowing mechanism which is valuable as single shot barium swallow may miss a small diverticulum [13]. Barium suspension video fluoroscopy was done on this case and clearly demonstrated the presence of proximal esophageal pouch and absence of hiatus hernia or reflux esophagitis.

ZD can be posterior, posterolateral, or lateral but the most common type is the posterior pulsion diverticulum [14]. Many authors classify the lesion according to size, measured in craniocaudal dimension. The diameter up to 2 cm is categorized as small, 2–4 cm—medium and 4–6 cm—large [15]. The diverticulum in this case was a posterior pulsion and was medium size (3.4 cm).

The differential diagnosis of esophageal diverticula depend up on the location of the diverticula [16]. Epiphrenic diverticula are usually located in the distal esophagus. The pathogenesis is considered to be secondary to esophageal motility disorders and is associated with congenital or acquired weakness of the esophageal wall [17]. The acquired area of weakness is known as Killian triangle or Killian’s dehiscence which is in the hypopharynx between two strong muscles—the inferior pharyngeal constrictor and cricopharyngeus muscles [18]. A continuous intraluminal pressure in this relative area of weakness leads to an outpouching and is the major pathogenesis of this entity [2, 19]. Diverticula of the middle esophagus were classified as Rokitansky diverticula, which commonly occur in the thoracic esophagus. They are believed to occur due to chronic inflammatory and fibrotic processes that pull the wall of the esophagus outward; consequently, they involve the three layers of the esophagus [20]. ZD are in the proximal esophagus and are pulsion diverticula [2].

The primary purpose of ZD management is, to alleviate symptoms and to improve the quality of life in which it was achieved in our patient [20]. ZD is managed surgically either by open left cervical incision or minimally invasive endoscopic approach [10]. Several surgical procedures have been reported: open diverticulectomy, rigid/flexible endoscopic diverticulotomy, diverticulopexy, diverticular inversion with or without myotomy, and myotomy alone [10, 20]. Endoscopic approach is gaining popularity due to its improved post-surgical morbidity of elderly patients [21]. The open surgical approach showed multiple disadvantages compared to endoscopic interventions especially in elderly and debilitating patients. The post-operative complication of open surgical approach in the general population was slightly higher than the flexible endoscopic approach (10.5% vs 8.7 %), and higher mortality rate of 0.6 versus 0.2% of endoscopic approach [10]. However, endoscopic approach showed no consistent long-term outcome in the literature with general estimated success rate of 63 to 100% and recurrence rate between 0 and 33% [10, 22]. Recurrent nerve injury, wound infection, hospital stay and fistula formation showed to reduce with endoscopic approach while intraoperative bleeding, and esophageal mucosal injury increased [23]. The patient was managed by flexible endoscopic diverticulotomy. There were no immediate or late post-operative complications.

In conclusion, ZD is a rare condition of the proximal esophagus, typically presents in elderly population. Barium swallow is the most important diagnostic tool in the diagnosis of ZD. Presenting symptoms can overlap with malignant conditions of the esophagus. High index of suspicion is needed to diagnose this condition as it is rare in the general population.

## Data Availability

The information used and/or analyzed during this case report is available from the corresponding author on reasonable request.

## Abbreviation

ZD: Zenker’s diverticulum
